# A case of a traumatic chyle leak following an acute thoracic spine injury: successful resolution with strict dietary manipulation

**DOI:** 10.1186/1749-7922-6-10

**Published:** 2011-03-28

**Authors:** Andrea M Pakula, Wendy Phillips, Ruby A Skinner

**Affiliations:** 1 Department of Surgery, Division of Trauma and Surgical Critical Care, Kern Medical Center, Bakersfield, California; 2 Department of Nutrition and Dietary Services, Kern Medical Center, Bakersfield, California

## Abstract

**Background:**

Chylothorax is a rare form of pleural effusion that can be associated with both traumatic and non-traumatic causes. Thoracic duct ligation is often the treatment of choice in postsurgical patients; however the optimal treatment of this disease process after traumatic injury remains unclear [1]. We present a rare case of a thoracic duct injury secondary to a blunt thoracic spine fracture and subluxation which was successfully treated non-operatively.

**Case Presentation:**

A 51 year old male presented as a tier one trauma code due to an automobile versus bicycle collision. His examination and radiographic work-up revealed fractures and a subluxation at the third and fourth thoracic spine levels resulting in paraplegia. He also sustained bilateral hemothoraces secondary to multiple rib fractures. Drainage of the left hemothorax led to the diagnosis of a traumatic chylothorax. The thoracic spine fractures were addressed with surgical stabilization and the chylothorax was successfully treated with drainage and dietary manipulation.

**Conclusions:**

This unusual and complex blunt thoracic duct injury required a multidisciplinary approach. Although the spine injury required surgical fixation, successful resolution of the chyle leak was achieved without surgical intervention.

## Introduction

The majority of reported cases of chylothorax are due to malignancy (50%) specifically non-Hodgkin's lymphoma. Chylothorax due to traumatic thoracic injuries including iatrogenic post surgical injuries comprise approximately twenty-five percent of cases. Other iatrogenic complications primarily related to central access catheters make up the remaining twenty-five percent [2,3]. This disease process, if not properly recognized and treated can lead to profound respiratory, nutritional and immunological dysfunction resulting in significant patient morbidity and mortality. The available treatment modalities include conservative management with drainage and strict dietary regulation or more invasive approaches namely thoracic duct ligation [4,5].

## Case Presentation

The patient is a 51 year old male who was struck by an automobile at 35 miles per hour while riding a bicycle. There was loss of consciousness in the field and he arrived to our level II trauma center in full spine precautions, as a tier one trauma code. His primary survey was intact and his initial vital signs were; BP 115/80, HR 84, RR 30, O2 saturation 89% on room air which improved to 98% on a non-rebreather mask at 100%. Pertinent findings on secondary survey revealed bilateral chest wall tenderness to palpation, diminished breath sounds bilaterally, upper thoracic spine tenderness to palpation, a complete loss of motor function in his lower extremities, a loss of sensory function below the level of T4 and a Glascow Coma Scale (GCS) of 15. His American Spine Injury Association Motor Score was 50. He also had a loss of his cremasteric reflex, and bulbar cavernous reflex, and had no sacral tone.

**Figure 1:**
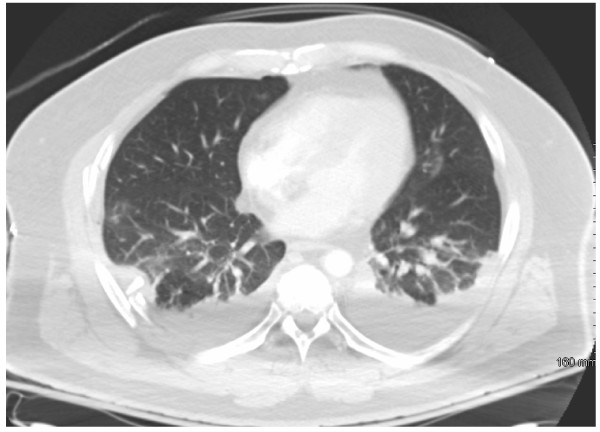
**CT scan of the chest illustrates bilateral pleural effusions**.

**Figure 2:**
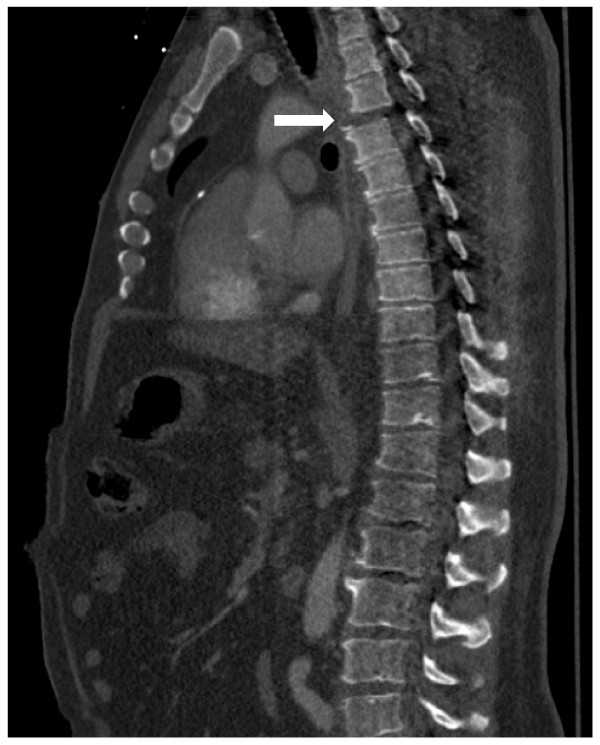
**Lateral CT scan of thoracic spine demonstrates T3/4 fracture dislocation (white arrow)**.

While observing strict spine precautions, the patient had chest and pelvis x-rays taken, and was then transported for computed tomography scans of the head, cervical spine and torso. Positive findings from these studies revealed multiple bilateral rib fractures with associated hemothoraces (Figure 1). He also sustained fractures and subluxation at the third and fourth thoracic levels (Figure 2). The patient was started on spinal dose steroids and strict spine precautions were maintained for anticipated surgical stabilization. Bilateral chest tube thoracostomies were placed for the hemothoraces and a arterial blood gas was then obtained which documented adequate oxygenation and ventilation given this patient's significant pulmonary injury; (pH 7.33 pCO_2 _42 PaO2 91 HCO3 21, O_2 _saturation 97 BD-4, 2 liters nasal cannula).

The initial drainage from the left chest tube was 500 milliliters (ml) of blood and on his second hospital day it was noted that the chest tube output was 400 ml of milky white fluid suspicious for chyle. Biochemical analysis of the pleural fluid revealed triglycerides of 287 milligrams/decilitre (mg/dL), total protein of 2600 mg/dL, and LDH of 2823 units/L. These results confirmed a diagnosis of chylothorax.

Due to the complexity of the case, a multidisciplinary team approach was taken to develop the appropriate treatment regimen for this patient. The decision to attempt treatment of the chyle leak with dietary manipulation was agreed upon and the patient was started on a very-low-fat oral diet consisting of mainly fresh fruits, vegetables and whole grains. The patient was also given a semi-elemental formula, Peptamen AF, 1 can with each meal which provided additional kilocalories, protein, and medium chain triglyceride (MCT) oil in order to facilitate wound healing. Two scoops of protein powder (beneprotein) were added to each meal as well. The patient was also started on octreotide, 200 mcg subcutaneous every 8 hours to aid in the reduction of lymph production. The patient tolerated the diet well and these measures led to a dramatic decrease in the chest tube output to less than 100 ml/day of serous fluid by the time he had operative repair and stabilization of his thoracic spine on hospital day seven. After the surgical procedure there was a transient increase in output from the chest tube to 200 ml per day which declined to 35 ml on hospital day 14. The chest tube was then removed without consequence, he was then started on a regular diet and follow up chest x-rays did not reveal any recurrent pleural effusions. The patient was discharged to an inpatient rehabilitation facility and was seen approximately two months after his injury in our clinic. He still had complete motor paralysis of the lower extremities with a T2 sensory loss. His upper extremity function remained unchanged from admission with his motor function intact. His pulmonary status remained stable as he had no ongoing acute pulmonary issues and saturated 98-100% on room air.

## Discussion

Chylous pleural effusion is most commonly caused by malignancy, accounting for more than 50% of cases. Lymphoma is the most common malignant cause, representing about 60% of all cases, with the non-Hodgkins variant being the most prevalent. Traumatic injuries to the upper abdomen and chest including those sustained during surgery are the second leading cause of chylothorax, accounting for approximately 25% of cases.

The first traumatic injury to the thoracic duct was described in 1875 and the first thoracic duct ligation was performed in 1948 [6]. The traumatic causes of injury to the duct vary widely, and the most common blunt mechanism producing injury is related to sudden hyperextension of the spine with rupture of the duct just above the diaphragm [4,7-9]. Sudden stretching over the vertebral bodies for any reason may tear the duct, but this usually occurs in the setting of a thoracic duct previously affected by disease [4,8]. Episodes of vomiting or a violent bout of coughing resulting in shearing of the lymphatic conduit along the crux of the right diaphragm has been reported as well [9]. Penetrating injuries, from a gunshot or stab wound, are less common and usually associated with severe damage to nearby structures.

The pertinent anatomy involved in the development of a chylothorax begins with the cysterna chyli, which is a confluence of lymphatics located in the retroperitoneum, just to the right of the posteromedial aorta at the level of the renal arteries. The thoracic duct ascends from this level and enters the chest through the aortic hiatus into the right hemithorax. The duct crosses over to the left chest at the fourth and fifth thoracic levels and enters the neck anterior to the left subclavian artery to join the venous system at the junction of the left subclavian vein and left internal jugular vein [10,11,13]. Knowledge of this anatomy should alert the physician to the possibility of a thoracic duct injury with thoracic spine fractures or any associated upper abdomen or chest injury involving this trajectory.

As in this case, the diagnosis of a chyle leak was supported by a pleural fluid triglyceride level greater than 110 mg/dL. A pleural fluid triglyceride concentration less than 50 mg/dL excludes a chylothorax. An intermediate level between 50 and 110 mg/dL should be followed by lipoprotein analysis to inspect the pleural fluid for chylomicrons or cholesterol crystals. The presence of chylomicrons and the absence of cholesterol crystals confirm a chyle leak. In addition, a ratio of pleural fluid cholesterol to triglyceride of less than 1 is also diagnostic [11,12].

Although most cases of traumatic chylothorax can be managed non-operatively, the need for surgical intervention in the subset of patients with associated thoracic fractures is higher and approaches 50 percent [5,11]. Indications for surgical intervention include an excessive leak for greater than five days, which for adults is > 500 ml/day and for children > 10 ml/kg/day or > 100 ml/day for each year of age. Any volume of output for greater than 14 days would indicate failure of conservative therapy and the need for surgical intervention as well [10].

Conservative treatment of chylothorax begins with prompt drainage via tube thoracostomy and dietary manipulation. The goal of dietary manipulation is to dramatically decrease lymph production since sixty percent of the dietary fat is absorbed by the lymphatic system, and approximately 1500 to 2000 ml of lymph is produced daily. This can be accomplished by completely bypassing the lymphatic circulation with Total Parenteral Nutrition (TPN), or by strict dietary manipulation based on a very low fat diet. We chose the latter to avoid some of the known infectious complications associated with TPN, especially in this high risk patient with multi-system trauma. With either approach, volume status, electrolyte abnormalities and nutritional parameters of the patient should be followed closely and aggressive replacement of nutritional losses of fat soluble vitamins and proteins should be carried out [10,14,15].

A diet strategy to limit chyle production involves avoidance of long chain triglycerides (LCTs). Medium chain triglycerides (MCTs), however, are absorbed directly into the portal circulation without stimulating lymphatic flow, so inclusion of these MCTs in the diet can help to increase caloric intake for healing [11]. These dietary changes as in our case were accomplished by a strict low fat diet supplemented with extra protein powder and MCT oil. Elemental peptide-based enteric formulas with less than 3% LCTs and MCTs added are also ideal supplements, as they have been shown to reduce the quantity and duration of chyle output. Though there is no consensus on the definitive duration of this nutritional management, the literature supports that these dietary measures be continued for approximately two weeks [10-12]. In general, once the chyle leak is resolved, then a regular diet may be resumed. In complicated cases, however, it may be advisable to ensure leak resolution with a provocative high-fat meal before removing a drainage tube.

Finally, there are anecdotal data supporting the use of octreotide to promote decreased lymphatic production. The mechanism is based on the reduction of gastrointestinal secretions and sphlancnic blood flow associated with octreotide, thus decreasing lymphatic production. The drug can be given as a continuous infusion or bolus injections [16,17]

## Conclusions

Although rare, traumatic chylothorax can be a difficult entity to manage especially in a patient with additional traumatic injuries. This case reflects a successful approach to the management of a traumatic chyle leak, using drainage and strict dietary changes, which precluded the need for surgical intervention.

## Consent

Written informed consent was obtained from the patient for publication of this case report and accompanying images. A copy of the written consent is available for review by the Editor-in-Chief of this journal.

## Competing interests

The authors declare that they have no competing interests.

## Authors' contributions

All authors contributed to researching, editing and writing the article. All authors read and approved the final manuscript.

## References

[B1] Maldonado F Medical and surgical management of chylothorax and associated outcomes Am J Med Sci 2010 339 4 314 318 20124878 10.1097/MAJ.0b013e3181cdcd6c

[B2] Doerr CH Allen MS Nichols FC Ryu JH Etiology of Chylothorax in 203 patients Mayo Clinic Proc 2005 80 7 867 870 10.4065/80.7.867 16007891

[B3] Fogli L Gorini P Belcastro S Conservative management of traumatic chylothorax: a case report Intens Care Med 1993 19 3 176 177 10.1007/BF01720537 8315128

[B4] Valentine V Raffin T The management of chylothorax Chest 1992 102 586 591 10.1378/chest.102.2.586 1643953

[B5] Paul S Surgical management of chylothorax Thorac Cardiovasc Surg 2009 57 4 226 228 10.1055/s-0029-1185457 19670117

[B6] Browse NL Allen DR Wilson NM Management of chylothorax Br J Surg 1997 84 12 1711 1716 10.1002/bjs.1800841218 9448622

[B7] Wright P Gardner A Traumatic chylothorax: A case after dislocation of the thoracic spine J Bone Joint Surg 1952 34B 64 67 10.1302/0301-620X.34B1.64 12999872

[B8] Brook M Dupree D Bilateral traumatic chylothorax Bilateral traumatic chylothorax 1988 17 69 72 10.1016/s0196-0644(88)80508-0 3122609

[B9] Biet AB Connolly NK Traumatic chylothorax; a report of a case and a survey of literature Br J Surg 1951 39 564 568 10.1002/bjs.18003915822 14935183

[B10] Spain DA The A.S.P.E.N. Nutrition Support Core Curriculum: A Case-Based Approach--The Adult Patient American Society of Parenteral and Enteral Nutrition 2007 477 487

[B11] Mason JF Murray JF, Nadel JA Chylothorax Murray and Nadel's Textbook of Respiratory Medicine 2010 5 Philadelphia: Saunders 1764 1792

[B12] Staats BA Ellefson RD Budahn LL Dines DE Prakash UB Offord UK The lipoprotein profile of chylous and nonchylous pleural effusions Mayo Clin Proc 1980 55 700 704 7442324

[B13] Miller JJ Shields T, Loccicero J, Ponn R, et al Anatomy of the thoracic duct & chylothorax General Thoracic Surgery 2005 6 Philadelphia: Lippincott, Williams & Wilkins 879 888

[B14] Sahn SA Pleural effusions of extra vascular origin Clin of Chest Med 2006 285 308 10.1016/j.ccm.2005.12.004 16716819

[B15] Apostolakis E Traumatic chylothorax following blunt trauma: two conservatively treated cases J Card Surg 2009 24 2 220 2 10.1111/j.1540-8191.2009.00828.x 19267837

[B16] Barili F Administration of octreotide for management of postoperative high-flow chylothorax Ann Vasc Surg 2007 21 1 90 92 10.1016/j.avsg.2006.02.001 17349344

[B17] Miura K Treatment of a persistent postoperative chylothorax with octreotide Kyobu Geka 2009 62 10 885 887 19764494

